# Dopamine, reward learning, and active inference

**DOI:** 10.3389/fncom.2015.00136

**Published:** 2015-11-04

**Authors:** Thomas H. B. FitzGerald, Raymond J. Dolan, Karl Friston

**Affiliations:** ^1^The Wellcome Trust Centre for Neuroimaging, University College LondonLondon, UK; ^2^Max Planck – UCL Centre for Computational Psychiatry and Ageing ResearchLondon, UK

**Keywords:** reward, reward learning, variational inference, dopamine, active inference, instrumental conditioning, incentive salience, learning

## Abstract

Temporal difference learning models propose phasic dopamine signaling encodes reward prediction errors that drive learning. This is supported by studies where optogenetic stimulation of dopamine neurons can stand in lieu of actual reward. Nevertheless, a large body of data also shows that dopamine is not necessary for learning, and that dopamine depletion primarily affects task performance. We offer a resolution to this paradox based on an hypothesis that dopamine encodes the precision of beliefs about alternative actions, and thus controls the outcome-sensitivity of behavior. We extend an active inference scheme for solving Markov decision processes to include learning, and show that simulated dopamine dynamics strongly resemble those actually observed during instrumental conditioning. Furthermore, simulated dopamine depletion impairs performance but spares learning, while simulated excitation of dopamine neurons drives reward learning, through aberrant inference about outcome states. Our formal approach provides a novel and parsimonious reconciliation of apparently divergent experimental findings.

## Introduction

Flexible and adaptive behavior requires, in many situations, that agents use explicit models of their environment to perform inference about the causes of incoming sensory information (Tenenbaum et al., 2006; Friston, 2010; Clark, 2012; Dolan and Dayan, 2013). When the structure of the environment is unknown, adaptive behavior requires agents address an additional challenge of learning the parameters of the models that they use. We consider learning in the particular context of active inference, an influential theory of decision-making, and action control. Active inference is based on a premise that agents choose actions using the same inferential mechanisms deployed in perception, with desired outcomes being simply those that an agent believes, *a priori*, that it will obtain (Friston et al., 2013).

**Figure 1:**
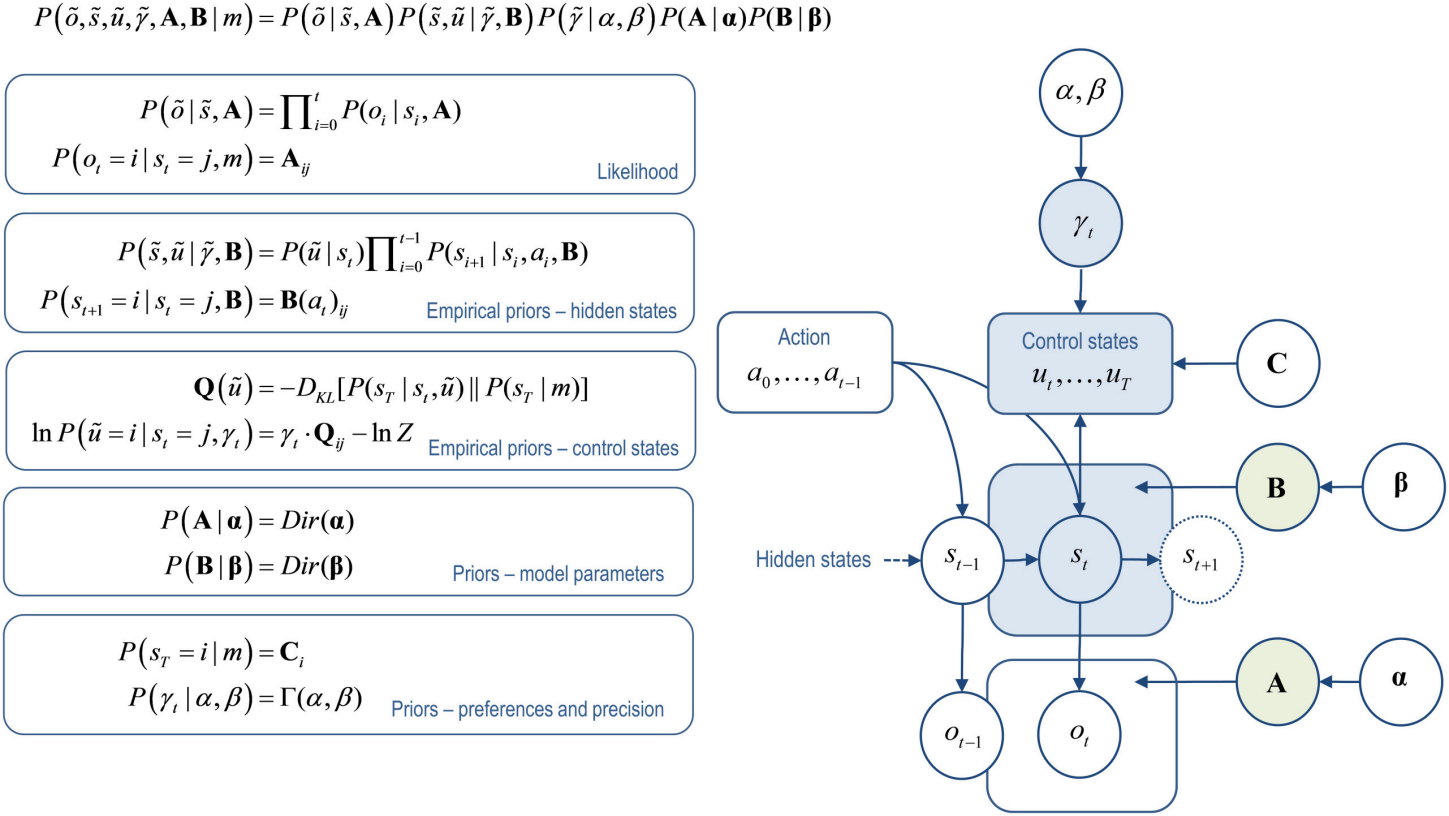
**Active inference model**. This illustrates dependencies between the variables in the augmented generative model of behavior (for further details see Friston et al., 2013). **Left**: these equations specify the generative model in terms of the joint probability over observations õ, hidden states $\tilde{s}$, control states ũ, the precision of beliefs about control states $\tilde{\gamma }$, and the parameters encoded by the matrices that determine the mapping between hidden states **A** and the transition probabilities between hidden states **B**(*u*). The form of these equations rests upon Markovian assumptions about controlled state transitions. **Right**: Bayesian graph showing the dependencies among hidden states and how they depend upon past and future control states. Sequences of future control states (policies) depend upon the current state, because policy selection depends upon the divergence between distributions over the final state that are, and are not, conditioned on the current state, together with the precision of beliefs about control states. Observed outcomes depend only on the hidden states at any given time. Given this generative model, an agent can make inferences about observed outcomes using variational Bayes (Beal, 2003). The same variational scheme can also learn the model parameters encoded by the A and B matrices. States and parameters are treated identically, except for the key distinction that because parameters are time-invariant, information about them can be accumulated over time. (States that are inferred upon are indicated in blue, parameters that are learnt in green).

Although, existing treatments of active inference largely assume that a model has already been learned (see Adams et al., 2012; Friston et al., 2012a,b; FitzGerald et al., 2015 for example), it is straightforward to incorporate learning within the same framework. We explore the consequences of learning under active inference, using a proposed framework for Markov decision processes (MDPs) and variational Bayes (Friston et al., 2013; Figure 1). This approach can elegantly simulate behavior on a number of tasks (Moutoussis et al., 2014; FitzGerald et al., 2015; Friston et al., 2015; Schwartenbeck et al., 2015a). Here it allows us to derive simple, generic, and biologically plausible learning rules for the parameters governing transitions between different hidden states of the world (see Equations 26–31 below), including linking hidden states with the observations they generate.

**Figure 2:**
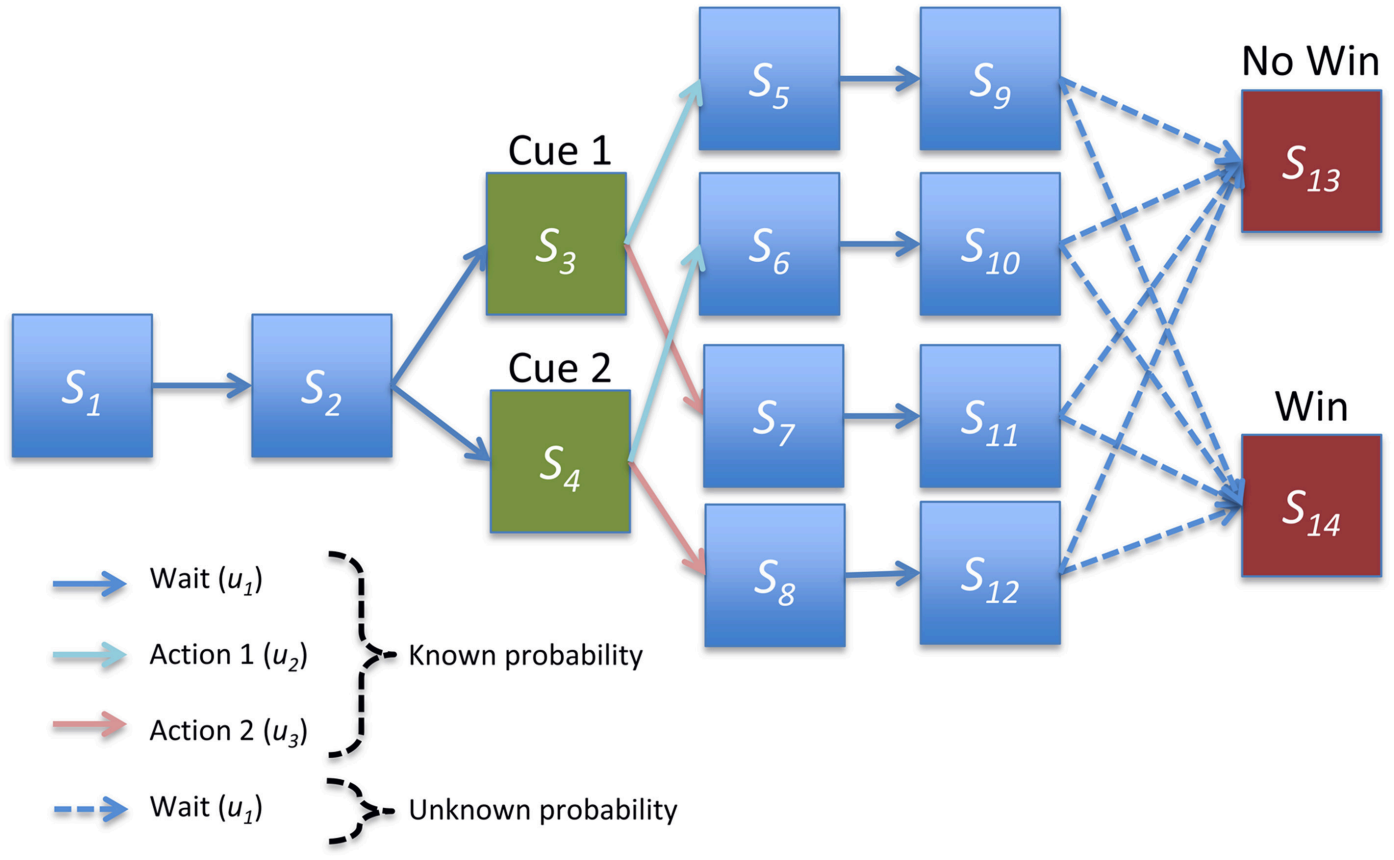
**Structure of instrumental conditioning task**. In each trial the agent first proceeds through two initial pre-cue states. One of two cues is then presented with equal probability, and the agent takes one of two actions. The agent then waits for two epochs or delayed periods, where each pair of hidden states corresponds to a particular cue-outcome combination. Finally, the agent moves probabilistically either to a win or no win outcome. Agents had strong and accurate beliefs about all transition probabilities except for the transitions to the final outcomes outcome, which had to be learnt.

We apply our augmented scheme to model instrumental conditioning (Figure 2), and show that it enables rapid and efficient learning. We then use this to test the plausibility of an hypothesis that dopamine encodes expected precision over control states (Friston et al., 2014; FitzGerald et al., 2015; Schwartenbeck et al., 2015a). Dopamine is a natural candidate for encoding expected precision for three reasons. First, because it exerts a modulatory (multiplicative) rather than driving effect on neuronal processing, in line with the role played by expected precision in our model. Second, because dopamine is the neuromodulator most closely linked to action and motivation, both in terms of the regions it innervates and what is known about its role in cognition. Third, because the dynamics of expected precision in response to rewards that naturally emerge from the model strongly resemble those of the phasic dopaminergic response.

**Figure 3:**
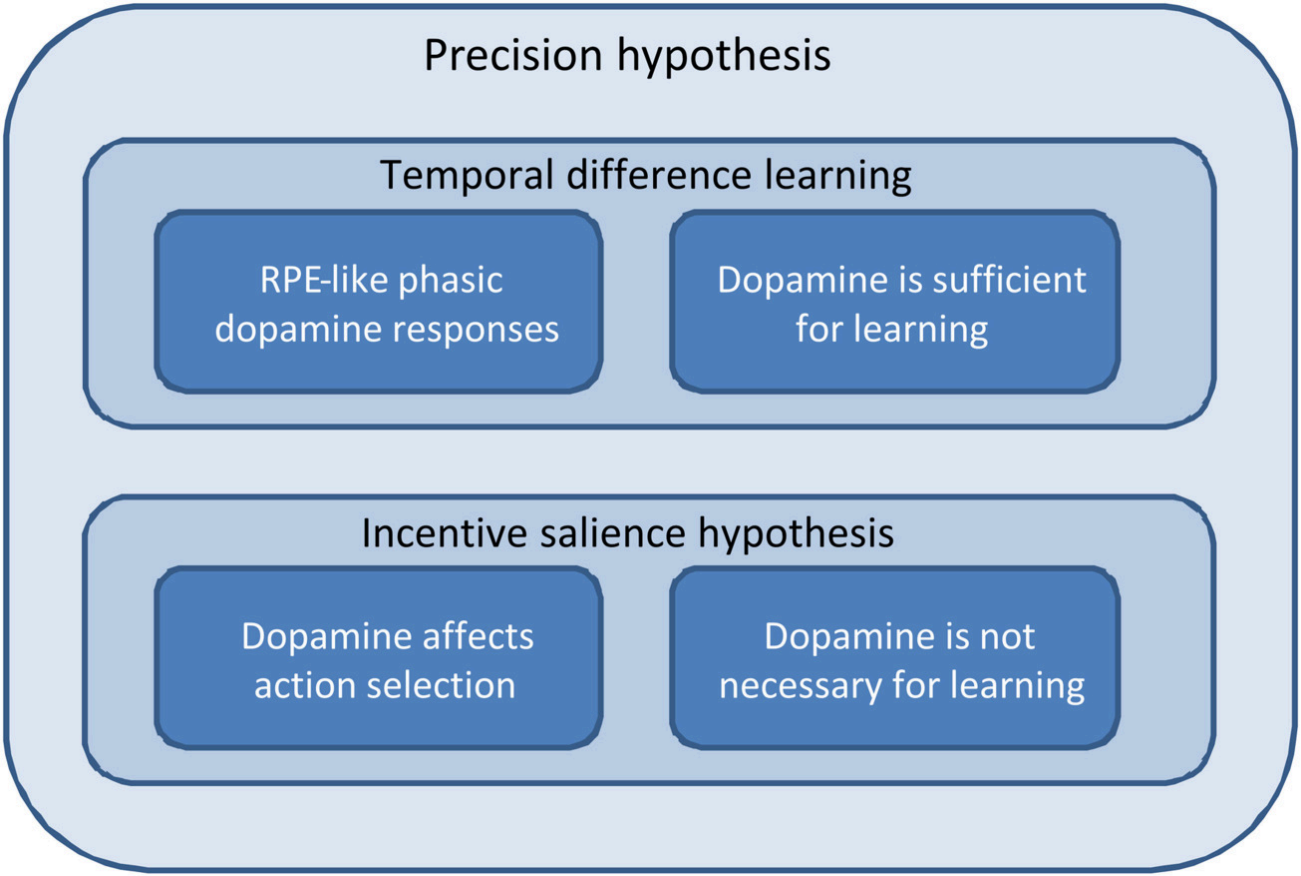
**Schematic depicting the relationships among temporal difference learning, incentive salience, and precision hypotheses; in terms of explaining the phenomena we consider in this paper**. The temporal difference learning hypothesis correctly predicts both reward prediction error-like phasic dopamine responses and the fact that dopaminergic stimulation is sufficient to establish preference learning. However, it does not predict either a direct effect of dopamine on action selection or the fact that dopamine is not necessary for preference learning. The incentive salience hypothesis, by contrast, predicts the effect of dopamine on action selection, and that it is not needed for learning, but struggles to explain the other two phenomena. The precision hypothesis, by contrast, accounts for all four. (This figure is intended to be illustrative rather than comprehensive, and we acknowledge that there are a number of key phenomena that are currently not well-explained by the precision hypothesis, as described in the Discussion).

To test this hypothesis, we reproduce three widely replicated empirical phenomena that, to our knowledge, are not adequately explained within any single extant normative theory (that is, one based on a presumed computational role for dopamine; Figure 3). The first concerns the acquisition of reward contingencies, known to induce characteristic changes in phasic activity in the dopaminergic system such that, over the course of learning, responses to rewarding outcomes are transferred to the stimuli which predict them (Schultz et al., 1997; Schultz, 1998; Day et al., 2007; D'Ardenne et al., 2008; Flagel et al., 2011; Cohen et al., 2012). This has led to the influential hypothesis that dopamine encodes reward prediction errors that underpin a form of temporal difference (TD) learning (Schultz et al., 1997). The second phenomenon we consider is the fact that although dopaminergic activity during learning is consistent with a reward learning prediction error (RPE), this disguises a puzzling fact that dopamine depletion primarily affects task performance rather than learning itself (Berridge and Robinson, 1998; Cannon and Palmiter, 2003; Robinson et al., 2005; Robbins and Everitt, 2007; Flagel et al., 2011; Berridge, 2012; Saunders and Robinson, 2012; Shiner et al., 2012; Smittenaar et al., 2012) [though see (Darvas and Palmiter, 2010)]. Here we acknowledge that although dopamine does not seem necessary for reward learning to occur, transient stimulation of dopaminergic midbrain neurons is sufficient to establish behavioral preference (Tsai et al., 2009; Adamantidis et al., 2011; Witten et al., 2011; Rossi et al., 2013; Steinberg et al., 2013; Stopper et al., 2014). This latter observation is our third explanandum.

We have highlighted a number of puzzling findings that are difficult to account for on either of the best established theories regarding the role of dopamine in motivated behavior. If dopamine encodes RPEs that drive learning (Schultz et al., 1997) then, *prima facie*, learning should be impaired in its near-absence. If on the other hand, dopamine encodes incentive salience (or “wanting”; Berridge, 2007), it is not clear why dopamine dynamics should so closely resemble RPEs on the one hand and why midbrain stimulation is sufficient to establish behavioral preference on the other, rather than just transiently motivating approach or consumption behavior. As such, explaining these three key findings within a unified framework would establish the precision hypothesis as a more parsimonious account of dopamine function.

## Materials and methods

### An active inference model for markov decision-processes

We first reprise our generic active inference scheme for solving MDPs (for details Friston et al., 2013). Briefly, the model considers series of observations {*o*_0_, …, *o*_*T*_} = õ that depend only upon hidden states $\left\{s_{0},\ldots ,s_{T}\right\}=\tilde{s}$. Transitions among hidden states are governed by sequences of control states {*u*_*t*_, …, *u*_*T*_} = ũ from the current time. These sequences constitute allowable policies π. Finally, actions are sampled from posterior beliefs over current control states. These beliefs are parameterized in terms of their confidence or precision $\left\{\gamma_{0},\ldots ,\gamma_{T}\right\}=\tilde{\gamma }$. Expectations about all states, including precision, are optimized to maximize model evidence or marginal likelihood (which is the same as minimizing surprise and variational free energy). In this setting, precision governs the stochasticity of behavior in a fashion analogous to the inverse temperature parameter of softmax decision rules, with the crucial difference that rather than being fixed, it is optimized in a context-sensitive fashion from moment to moment.

In this model, future control states or policies depend upon the current hidden state, because the probability that the agent assigns to different policies rests upon their value or quality *Q*(ũ) = −*D*_*KL*_[*P*(*s*_*T*_ | *s*_*t*_, ũ) || *P*(*s*_*T*_ | *m*)]. This corresponds to the (negative) Kullback-Leibler (KL) divergence between distributions over the final state that are, and are not, informed by the current state. (Here, *m* indicates the agent's generative model) In other words, policies are considered more likely when they minimize the difference between the predictive distribution over final states, given the current and preferred states encoded by prior beliefs. This provides a fairly generic form of risk sensitive or KL control.

Under this scheme, a generative model is specified completely with three matrices, (and hyperparameters governing precision): the observation matrix **A** constitutes the parameters of the likelihood model and encodes the probability of an outcome, given a hidden state. The second set of matrixes, **B**(*u*) specify probabilistic transitions between hidden states that depend on the current control state. Lastly, the vector **C** encodes the prior probability of—or preference for—different terminal states *C*(*s*_*T*_) = ln *P*(*s*_*T*_ | *m*), where the logarithm of this probability corresponds to the utility of each final (hidden) state. Previously, we have considered inference problems where these matrices are assumed to be known (Friston et al., 2013; FitzGerald et al., 2015). However, by treating the **A** and **B** matrices as encoding unknown parameters, exactly the same scheme can be augmented to include learning, as described below.

### Variational learning in active inference

A key plank of the active inference scheme described above (and variational methods more generally) is a mean field assumption. This approximates the joint distribution over a set of variables by assuming conditional independence among subsets to render Bayesian model inversion analytically tractable (Bishop, 2006). With a careful choice of prior distributions, it is possible to perform (approximately) optimal inference by iteratively evaluating the variables in each subset in terms of the sufficient statistics of the other subsets, a procedure known as variational Bayes (Beal, 2003). This scheme is fast and depends only upon simple message passing between different subsets of unknown variables. It thus, constitutes a plausible metaphor for neuronal implementations of Bayesian inference (Friston et al., 2013).

To include learning within our variational scheme we simply add extra variables, corresponding to the model parameters to be learnt. The important difference between states and parameters is that parameters are time-invariant, whereas states are not. This means that information about parameters is accumulated across trials leading to a progressive minimization of (average) surprise as the structure of the environment is learned. In this setting, *inference* corresponds to optimizing expectations about hidden states of the world generating outcomes, while *learning* refers to the optimization of the parameters of the underlying generative model. In short, simply by including parameters in a variational update scheme, we can seamlessly incorporate learning within active inference.

Many different realizations of variational learning are conceivable, which will vary in their efficacy and biological plausibility. In particular, a key difference is whether learning takes place only “online” using currently available information, or whether additional “offline” learning occurs using information gathered during some extended period of time as, for example, when complete experimental trial or run through a maze also occurs. This corresponds to the difference between Bayesian filtering and smoothing, and the possibility of forward and backward sweeps during approximate Bayesian inference (see Penny et al., 2013). Here, we consider online learning, which, as will be seen below, has a natural resemblance to Hebbian learning schemes (Abbott and Nelson, 2000), and thus *prima facie* embodies a neurobiological plausibility.

### Augmenting the generative model

In this paper, we consider learning the parameters of the observation matrix **A** that maps from hidden states to observations, and the state transition matrices **B**(*u*) that map from the current hidden state to the next state. The **A** matrix comprises a set of multinomial probability distributions in each column. This means the *j*-th column of the observation matrix **A**_∙*j*_ encodes the likelihood of different observations, given the current hidden state. Since the conjugate prior of the multinomial distribution is the Dirichlet distribution, it is convenient to place a Dirichlet prior over each of these multinomial distributions, with concentration parameters **α** such that:

(1) $$\begin{array}{l} P\;\left(o_{t}=i|s_{t}=j,m\right)={\text{A}}_{ij} \end{array}$$

(2) $$\begin{array}{l} P\;\left({\text{A}}_{∙j}|\text{α}\right)=Dir\left({\text{α}}_{∙j}\right) \end{array}$$

(3) $$\begin{array}{l} \ln\;E_{P}\left[{\text{A}}_{ij}\right]=\ln\;\left({\text{α}}_{ij}\right)-\ln\;\left({\text{α}}_{j}^{0}\right) \end{array}$$

(4) $$\begin{array}{l} E_{P}\left[\ln\;{\text{A}}_{ij}\right]=\psi \left({\text{α}}_{ij}\right)-\psi \left({\text{α}}_{j}^{0}\right) \end{array}$$

(5) $$\begin{array}{l} {\text{α}}_{j}^{0}=\sum_{i}{\text{α}}_{ij} \end{array}$$

Here, we have included expressions for the log of the expected probability and the expected log probability, where ψ(·) is the digamma or psi function. These expressions will be important later, when we examine the corresponding posterior distributions (which are also Dirichlet distributions, because the priors are conjugate to the likelihood). Similarly, each **B**(*u*) matrix encodes a set of multinomial probability distributions mapping from current states to immediate future states.

(6) $$\begin{array}{l} P\;\left(s_{t+1}=i|s_{t}=j,u,\text{B}\right)=\text{B}{\left(u\right)}_{ij} \end{array}$$

(7) $$\begin{array}{l} P\;\left(\text{B}{\left(u\right)}_{∙j}|\text{β}\left(u\right)\right)=Dir\left(\text{β}{\left(u\right)}_{∙j}\right) \end{array}$$

With these priors in place, we now consider how the parameters are learnt.

### Learning and free energy

From a purely formal standpoint, learning should progressively reduce average surprise or maximize the accumulated evidence for a generative model. In the context of variational learning, surprise is conveniently approximated by the variational free energy which is minimized during learning and inference. The free energy can be expressed as a function of observations and the sufficient statistics (e.g., expectations) of an approximate posterior distribution defined by the mean field assumption. Let, $\tilde{x}=\tilde{s},ũ,\tilde{\gamma },\text{A},\text{B}$ denote the hidden variables and $\overset{⌢}{x}=\overset{⌢}{s},\overset{⌢}{\pi },\overset{⌢}{\gamma },\overset{⌢}{\text{α}},\overset{⌢}{\text{β}}$ the sufficient statistics of an approximate posterior distribution $Q\;\left(\tilde{x}|\overset{⌢}{x}\right)$ we want to optimize with respect to free energy, which can be written as (with a slight abuse of notation):

(8) $$\begin{array}{l} F_{t}={\text{E}}_{Q}\left[\ln\;P\left(o_{t}|\tilde{x}\right)\right]-D_{KL}\left[Q\left(\tilde{x}|\overset{⌢}{x}\right)||P\left(\tilde{x}|m\right)\right] \end{array}$$

(9) $$\begin{array}{l} P\;\left(o_{t},\tilde{x}|m\right)=P\left(o_{t}|\tilde{s},\text{A}\right)P\;\left(\tilde{s},ũ|\tilde{\gamma },\text{B}\right)\\ P\left(\gamma |\alpha ,\beta \right)P\left(\text{A}|\text{α}\right)P\left(\text{B}|\text{β}\right)\; \end{array}$$

(10) $$\begin{array}{l} P\;\left(\tilde{\gamma }|\alpha ,\beta \right)=\Gamma \left(\alpha ,\beta \right) \end{array}$$

(11) $$\begin{array}{l} P\;\left(\text{A}|\text{α}\right)=Dir\left(\text{α}\right) \end{array}$$

(12) $$\begin{array}{l} P\left(\text{B}|\text{β}\right)=Dir\left(\text{β}\right) \end{array}$$

(13) $$\begin{array}{l} Q\left(\tilde{x}|\overset{⌢}{x}\right)=Q\left(s_{t}|{\overset{⌢}{s}}_{t}\right)Q\left(ũ|\overset{⌢}{\pi }\right)\\ Q\left(\tilde{\gamma }|\overset{⌢}{\gamma }\right)Q\left(\text{A}|\overset{⌢}{\text{α}}\right)Q\left(\text{B}|\overset{⌢}{\text{β}}\right) \end{array}$$

(14) $$\begin{array}{l} Q\left(\tilde{\gamma }|\overset{⌢}{\gamma }\right)=\Gamma \left(\alpha ,\overset{⌢}{\beta }=\alpha ∕\overset{⌢}{\gamma }\right) \end{array}$$

(15) $$\begin{array}{l} Q\;\left(\text{A}|\overset{⌢}{\text{α}}\right)=Dir\left(\overset{⌢}{\text{α}}\right) \end{array}$$

(16) $$\begin{array}{l} Q\;\left(\text{B}|\overset{⌢}{\text{β}}\right)=Dir\left(\overset{⌢}{\text{β}}\right) \end{array}$$

The first equality (8) expresses free energy in terms of the accuracy or expected log likelihood of the current observation and a complexity term. This complexity term is the KL divergence between the approximate posterior and prior distributions. The second set of equalities (9–12) includes our Dirichlet priors over the unknown parameters, while the third set of equalities (13–16) specifies our mean field assumption and the form of its marginal distributions (induced by our use of conjugate priors). One can now express inference and learning as a minimization of accumulated free energy, which can be nicely expressed in terms of Action $S\left(õ,\overset{⌢}{x}\right)$ or the path integral of free energy, so that inference and learning conform to Hamilton's principle of least action:

(17) $$\begin{array}{l} S\left(õ,\overset{⌢}{x}\right)=\sum_{t}F_{t}\left(o_{t},\overset{⌢}{x}\right) \end{array}$$

(18) $$\begin{array}{l} {\overset{⌢}{s}}_{t}^{*}={\arg\min}_{{\overset{⌢}{s}}_{t}}\;S\left(õ,\overset{⌢}{x}\right)={\arg\min}_{{\overset{⌢}{s}}_{t}}\;F_{t}\left(o_{t},\overset{⌢}{x}\right) \end{array}$$

(19) $$\begin{array}{l} {\overset{⌢}{\pi }}^{*}={\arg\min}_{\overset{⌢}{\pi }}\;S\left(õ,\overset{⌢}{x}\right)={\arg\min}_{\overset{⌢}{\pi }}\;F_{t}\left(o_{t},\overset{⌢}{x}\right) \end{array}$$

(20) $$\begin{array}{l} {\overset{⌢}{\gamma }}^{*}={\arg\min}_{\overset{⌢}{\gamma }}\;S\left(õ,\overset{⌢}{x}\right)={\arg\min}_{\overset{⌢}{\gamma }}\;F_{t}\left(o_{t},\overset{⌢}{x}\right) \end{array}$$

(21) $$\begin{array}{l} {\overset{⌢}{\text{α}}}^{*}={\arg\min}_{\overset{⌢}{\text{α}}}\;S\left(õ,\overset{⌢}{x}\right) \end{array}$$

(22) $$\begin{array}{l} \overset{⌢}{\text{β}}{\left(u\right)}^{*}={\arg\min}_{\overset{⌢}{\text{β}}\left(u\right)}\;S\left(õ,\overset{⌢}{x}\right) \end{array}$$

Note that inference Equations (18–20) only needs to minimize free energy at the current time point, while learning Equations (21, 22) accumulates information over time. With the generative model and mean field assumption above, it is straightforward to solve for the sufficient statistics that minimize free energy, leading to the following variational updates (see Appendix and Beal, 2003)

(23) $$\begin{array}{l} {\overset{⌢}{s}}_{t}=\text{σ}\left(\overset{⌢}{\text{A}}\cdot o_{t}+\overset{⌢}{\text{B}}\left(a_{t-1}\right){\overset{⌢}{s}}_{t-1}+\overset{⌢}{\gamma }\cdot \text{Q}\cdot \overset{⌢}{\pi }\right) \end{array}$$

(24) $$\begin{array}{l} \overset{⌢}{\pi }=\sigma \left(\overset{⌢}{\gamma }\text{Q}{\overset{⌢}{s}}_{t}\right) \end{array}$$

(25) $$\begin{array}{l} \overset{⌢}{\gamma }=\frac{\alpha }{\beta -\overset{⌢}{\pi }\cdot \text{Q}{\overset{⌢}{s}}_{t}} \end{array}$$

(26) $$\begin{array}{l} {\overset{⌢}{\text{A}}}_{ij}=\psi \left({\overset{⌢}{\text{α}}}_{ij}\right)-\psi \left({\overset{⌢}{\text{α}}}_{j}^{0}\right) \end{array}$$

(27) $$\begin{array}{l} {\overset{⌢}{\text{α}}}_{ij}={\text{α}}_{ij}+\sum_{t}o_{ti}{\overset{⌢}{s}}_{tj} \end{array}$$

(28) $$\begin{array}{l} {\overset{⌢}{\text{α}}}_{j}^{0}=\sum_{i}{\overset{⌢}{\text{α}}}_{ij} \end{array}$$

(29) $$\begin{array}{l} \overset{⌢}{\text{B}}{\left(u\right)}_{ij}=\psi \left(\overset{⌢}{\text{β}}{\left(u\right)}_{ij}\right)-\psi \left(\overset{⌢}{\text{β}}{\left(u\right)}_{j}^{0}\right) \end{array}$$

(30) $$\begin{array}{l} \overset{⌢}{\text{β}}{\left(u\right)}_{ij}=\text{β}{\left(u\right)}_{ij}+\sum_{t}\left[u=a_{t-1}\right]\cdot {\overset{⌢}{s}}_{ti}{\overset{⌢}{s}}_{t-1j} \end{array}$$

(31) $$\begin{array}{l} {\overset{⌢}{\text{β}}}_{j}^{0}=\sum_{i}{\overset{⌢}{\text{β}}}_{ij} \end{array}$$

Here, the Iverson brackets [·] returns one if the expression is true and zero otherwise, and here it ensures the appropriate state-transition matrix is updated following a particular action. Iterating these updates provides Bayesian estimates of the unknown variables. This means that the sufficient statistics change over two timescales: a fast timescale that updates posterior beliefs between observations and a slow timescale that updates posterior beliefs as new observations are sampled. We now consider each update in turn:

The first Equation (23) updates expectations about hidden states and corresponds to *perceptual inference* or *state estimation*. This is essentially a Bayesian filter that combines predictions based upon expectations about the previous state with the likelihood of the current observation. The last term in the first equality represents an *optimism bias* that biases perception toward those hidden states that have the greatest value, those expected under beliefs about the policy. This will play an important role later when we simulate false inference by fixing expected precision at higher levels.

The second update Equation (24) is just a softmax function of the expected value of each policy under the inferred current state. Here, the sensitivity parameter or expected precision is an increasing function of expected value. This means that the sensitivity or inverse temperature, that determines the precision with which a policy selected, increases with the expected value of those policies. The third update Equation (25) optimizes the expected precision of beliefs over policies, such that if an observation increases the expected value of the policies, then expected precision increases and the agent is more confident in selecting the next action. This may explain why dopamine discharges have been interpreted in terms of changes in expected value (e.g., reward prediction errors). The role of dopamine in encoding precision is motivated easily by noting that precision enters the belief updates in a multiplicative or modulatory fashion.

The last two update rules Equations (26–31) for the parameters differ markedly in form from the inference and bear a marked resemblance to classical Hebbian plasticity (Abbott and Nelson, 2000). Each comprises two terms: an associative term that is a digamma function of the accumulated product of expected (postsynaptic) outcomes and their (presynaptic) causes and a decay term that reduces each connection as the total input connectivity increases. The associative and decay terms are strictly increasing but saturating (digamma) functions of the concentration parameters. Note that the ensuing updates do not have an explicit learning rate: the learning rate is implicitly determined by the sum of the concentration parameters (see Equations 1–5). This sum depends upon the number of observations on which the agent's beliefs are based. Thus, reassuringly, the larger the number of observations, the less they will be altered by new information. Intuitively, learning about the observation matrix depends upon coincident firing of presynaptic neurons encoding *s*_*t*_ and postsynaptic neurons encoding *o*_*t*_. In a similar fashion, learning about the state transition matrices depends upon firing in neurons encoding the previous state *s*_*t*−1_ that coincides with firing in neurons encoding the current state *s*_*t*_.

Neurobiologically, it seems plausible to distinguish between rapidly changing neuronal activity that encodes states, and the (slower) process of synaptic plasticity, which is likely to mediate learning. From a formal perspective, an interesting feature of this (Bayes-optimal) variational learning is that expected precision acts vicariously through its modulatory effects on the expected states. Mathematically, this is because the sufficient statistics of the parameters and the precision are separated by a Markov blanket (see Figure 1). This means the parameter updates are not a function of expected precision. Neurobiologically, one would interpret this conditional independence as an effect of dopamine on learning that is mediated entirely through its neuromodulatory effects on postsynaptic responses. If we associate precision with dopamine, one would have to conclude that dopamine *does not* play the role of a teaching signal that enables associative plasticity—it simply modulates postsynaptic responses that drive activity-dependent learning. An unavoidable prediction here is that it should be impossible to induce reinforcement learning or synaptic plasticity by stimulating dopaminergic firing in the absence of any postsynaptic depolarization. Conversely, in the absence of dopamine both state estimation and learning should proceed, with the only difference being a loss of optimism bias and confident (precise) action selection. This contrasts with extant (dopamine as a teaching signal or reward prediction error) formulations, which predict that no learning should occur in the absence of dopamine.

In principle, this scheme could be further augmented to encode learning about other parameters, including the hyperparameters encoding prior beliefs about precision which might be used, for example, to explain the relationship between average reward rate and vigor (Beierholm et al., 2013). For simplicity, we do not deal with this here, but will treat it in future work.

### Simulations of learning and instrumental conditioning

We applied the generic scheme described above to model behavior during a simple instrumental conditioning task in which the agent is presented with one of two cues and can make one of two responses. Each cue-response combination led to a reward with some fixed probability. To examine the transfer of precision from rewards to cues, we used a trace conditioning paradigm and included states corresponding to delay periods. This resulted in 14 hidden states, with two initial waiting (pre-cue) states, two cue states, four pairs of delay period states (one pair each of the four cue-response combinations), and two outcome states (“win” and “no win”; Figure 2). The three control states entail doing nothing or taking one of two actions after cue presentation on the third epoch of each trial (these might correspond, for example, to pressing one of two levers).

The generative process governing transitions between states is illustrated in Figure 2. Briefly, the agent always begins in the first pre-cue state and moves deterministically to the second. One of two cues (i.e., conditioned stimuli) are then presented with equal probability, and the agent then progresses through delay period states, corresponding to each cue-response combination, before making a stochastic transition to one of the two outcome states. Because there are separate delay periods for each cue-response combination, the agent effectively remembers what has happened and what it has done. However, it does not know the consequences of its choices until the final (outcome) state. It is these consequences the agent has to learn, solving the temporal credit assignment problem through implicit memory (i.e., with perceptual inference).

All transition probabilities were known to a high degree of certainty by the agent (with large concentration parameter values for deterministic transitions) apart from the final transition to the outcome states (Figure 2). These were given weak initial priors with concentration parameters of 1 on the transition to the no-win outcome state, 0.4 on the transition to a win outcome state, and negligible values for other transitions. Intuitively, this corresponds to a weak prior that each cue-response combination is unlikely to lead to reward. Learning corresponds to updating these prior beliefs by accumulating evidence for the actual reward contingencies.

The mapping between hidden states and observations allowed for five possible observations, a single observation generated deterministically in the initial and delay period states (corresponding to nothing happening, indicated by blue boxes in Figure 2), two observations corresponding to the two possible cues (indicated by green boxes), and two outcome observations (indicated by red boxes). Unless otherwise specified, we assume that agents have strong and accurate prior beliefs about the parameters of the observation matrix, enabling us to focus on learning of the (choice dependent) transitions to the final outcome.

**Figure 4:**
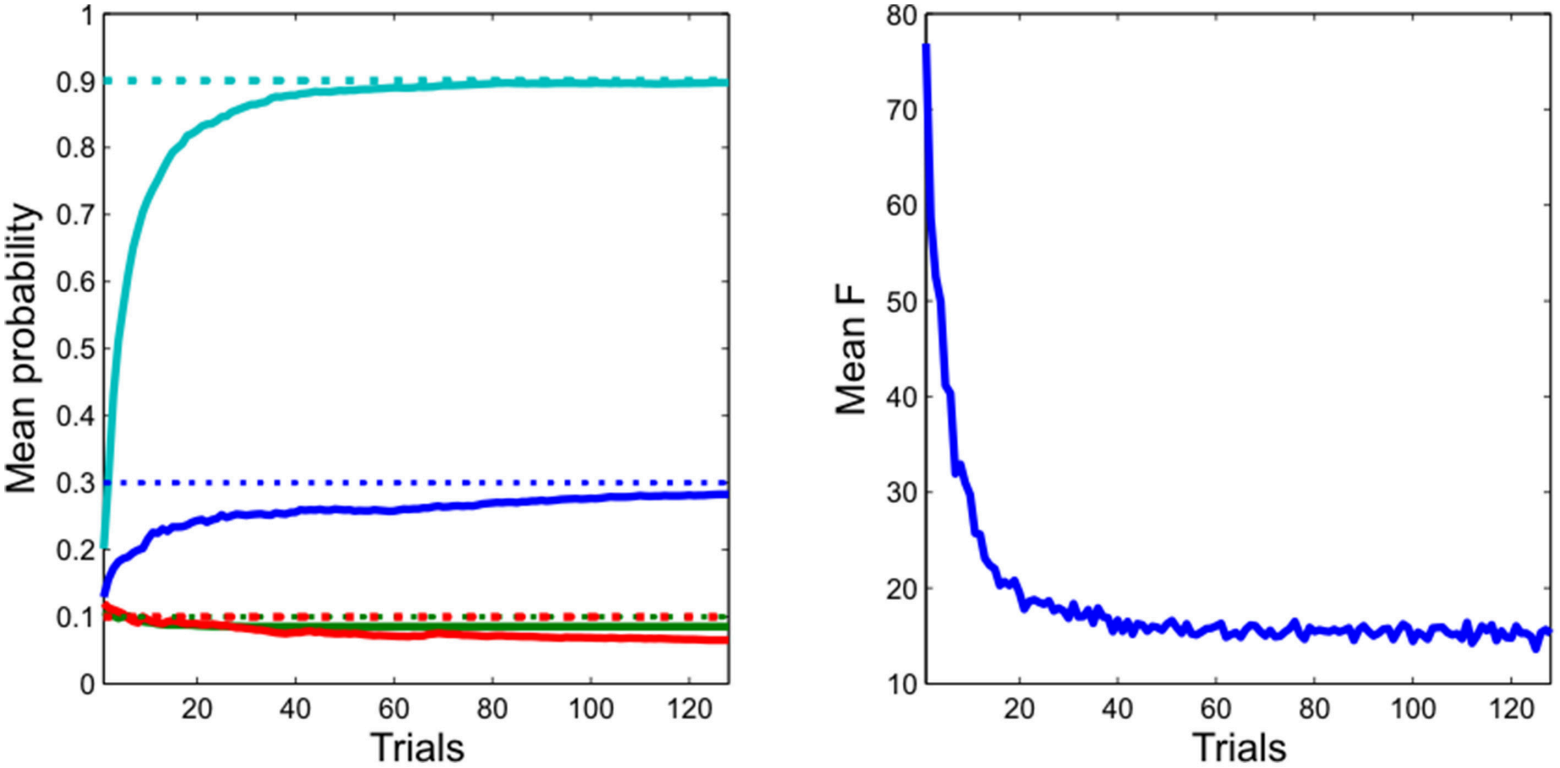
**Learning performance**. **Left**: the agent rapidly and accurately learns the unknown transition probabilities. (Dotted lines: actual values, continuous lines: estimated values) **Right**: this is accompanied by a progressive reduction in the variational free energy, confirming the agent has improved its model of the task. (Data are averaged across 256 repetitions of 128 simulated trials).

**Figure 5:**
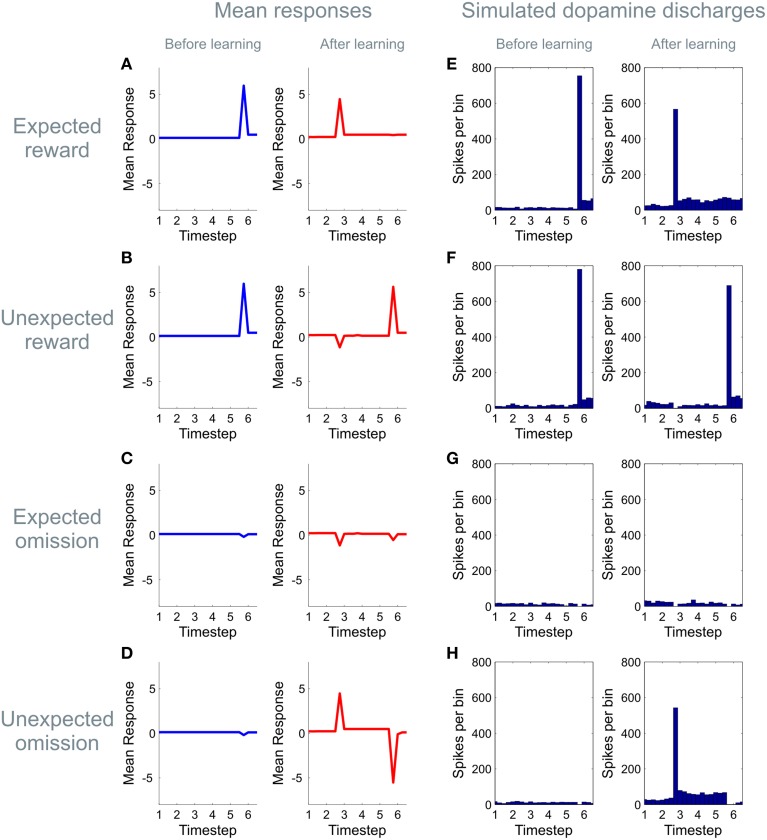
**Learning induced changes in the dynamics of the dopamine signal**. The panels on the left hand side of the figure **(A–D)** show simulated dopaminergic dynamics at a population level, whilst those on the right hand side **(E–H)** show simulated activity in dopaminergic neurons assuming that an expected precision of one is encoded by four spikes per bin with a background firing rate of four spikes per bin. (Firing rates are simulated using a Poisson process, averaged over 64 simulated trials) Here we illustrate simulated dopamine responses for four trial types, those on which a cue predicting a high likelihood of reward is presented and a reward is received (“expected reward,” **A,E**), or omitted (“unexpected omission,” **D,H**), and those on which a cue predicting a low likelihood of reward is presented, and a reward is received (“unexpected reward,” **B,F**) or omitted (“expected omission,” **C,G**). (For details of the simulations, see main text) Before learning (blue), no expectations have been established, and dopamine responses to reward-predicting stimuli are absent (time point three), but clear responses are shown to rewarding outcomes (time point six, top two rows). (The small dip when reward is omitted (bottom two rows) reflects the agent's initial belief that it will receive reward with a small but non-zero probability at the end of each trial). After learning (red), by contrast, clear positive responses are seen to the high reward cue (top and bottom rows) with a dip accompanying the presentation of the low-reward cue (middle rows). Learning also induces changes in the responses to outcomes, such that when reward is strongly expected responses to rewarding outcomes are strongly attenuated **(A,E)**, and those to reward omissions increased **(D,H)**. This mirrors the “reward prediction error” pattern of responding widely reported to occur in dopamine neurons during conditioning.

**Figure 6:**
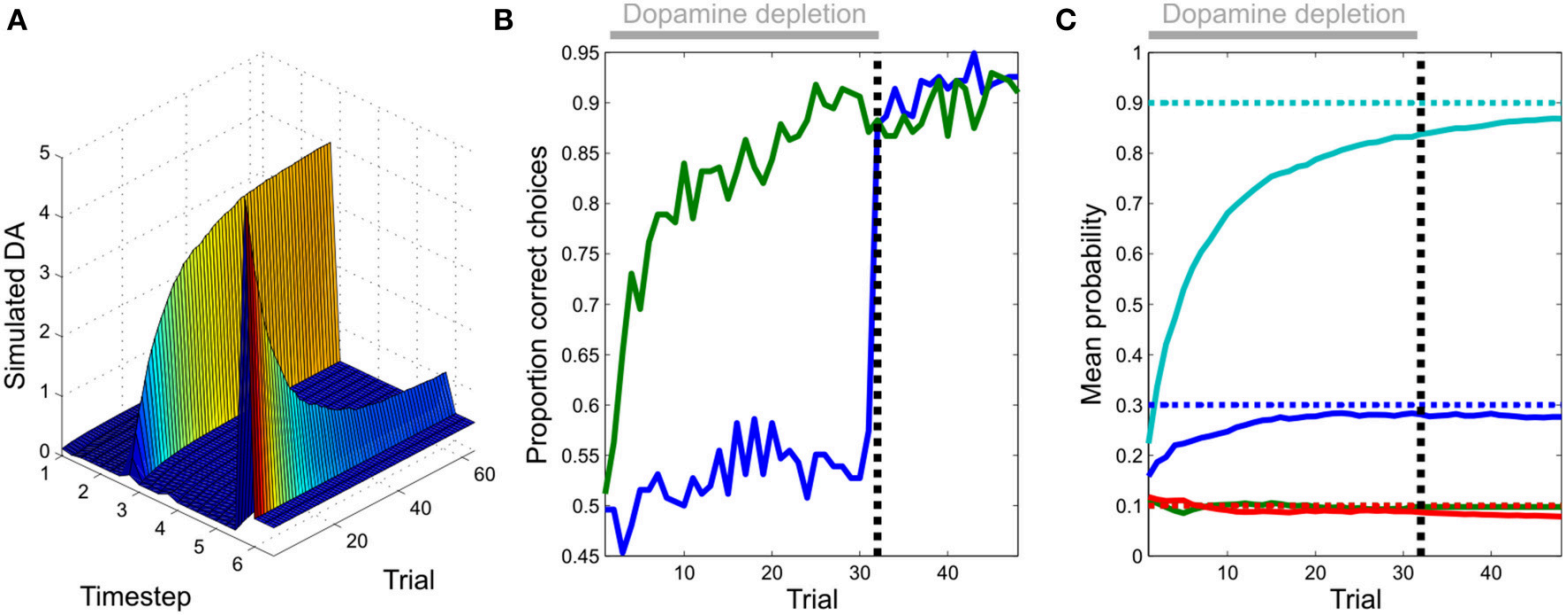
**Evolution of dopamine responses and effects of dopamine on behavior**. **(A)** Transfer of simulated dopamine responses from outcome to cue during learning. Responses to rewarding outcomes (epoch or update six) diminish over the course of learning, whilst those to the reward-predicting cue (epoch three) increase in magnitude. Unlike in many temporal difference learning models, the transfer of responses is direct (i.e., not mediated by dopamine responses at intervening time points). This constitutes a clear and testable prediction of our model, when compared with temporal difference learning accounts of phasic dopamine responses. **(B)** The effects of simulated dopamine depletion on task performance. Fixing expected precision to a low value (0.1) appears to prevent learning, as indexed by the proportion of correct responses selected by the agent (blue line, first 32 trials). However, learning does in fact occur, but is simply masked by the effects of reduced precision on choice behavior. This is revealed after restoration of normal function (trial 33 onwards), at which point performance becomes comparable to that of a non-lesioned agent. (Figure shows choice behavior averaged across 256 simulated sessions). **(C)** Parameter learning during dopamine depletion. The agent is able to accurately learn unknown transition probabilities as during normal function (Figure 4), even though this is masked by the effects of dopamine on action selection as shown in **(B)** (Dotted lines: actual values, continuous lines: estimated values).

To test the performance of our learning scheme we simulated 256 repetitions of 128 trials (each comprising six epochs or state transitions), and tracked how well, on average, the agent learnt the transition probabilities to the outcome states (and thus the correct instrumental contingencies; Figure 4). We also calculated the average free energy, which should progressively decrease over the course of learning. To simulate dopamine before and after learning, we simulated responses to four fixed trial types. These corresponded to the agent observing both possible cues and getting both possible outcomes, using both the “naïve” (pre-learning) parameters, and those from a randomly selected learning session (Figure 5). To make these exactly comparable, we only consider trials when the agent chose the rewarded option. To characterize the evolution of dopaminergic responses, we simulated responses to a single trial type (where the high reward cue was presented, the correct action was selected, and reward was received), using the model parameters for the first 64 trials, averaged across all sessions (Figure 6A).

Dopamine responses themselves were simulated by de-convolving the variational updates for expected precision by an exponentially decaying kernel with a time constant of 16 iterations. In other words, we assume that dopamine increases expected precision, which subsequently decays the time constant of 16 updates. To illustrate the sort of empirical responses one might see, we also simulated histograms by assuming a Poisson discharge rate of four spikes per bin corresponds to an expected precision of unity, with a background firing rate of four spikes per bin. Histograms were averaged across 64 simulated trials.

### Simulating dopamine depletion

To model the effects of dopamine deletion on task performance and learning we fixed precision to a low value $\left(\overset{⌢}{\gamma }=0.1\right)$ throughout all six epochs or updates and simulated 256 repetitions of 32 trials (Figure 6B). We compared the average number of correct decisions (defined as selecting the action objectively most likely to lead to reward on each trial) made by the “dopamine depleted” agent, with that made by a normal agent (one in which precision was updated normally as described above). To simulate the effects of dopamine restoration after learning, we restored normal precision updates to the previously dopamine-depleted agent and simulated 256 repetitions of a further 16 trials.

### Simulating midbrain stimulation

**Figure 7:**
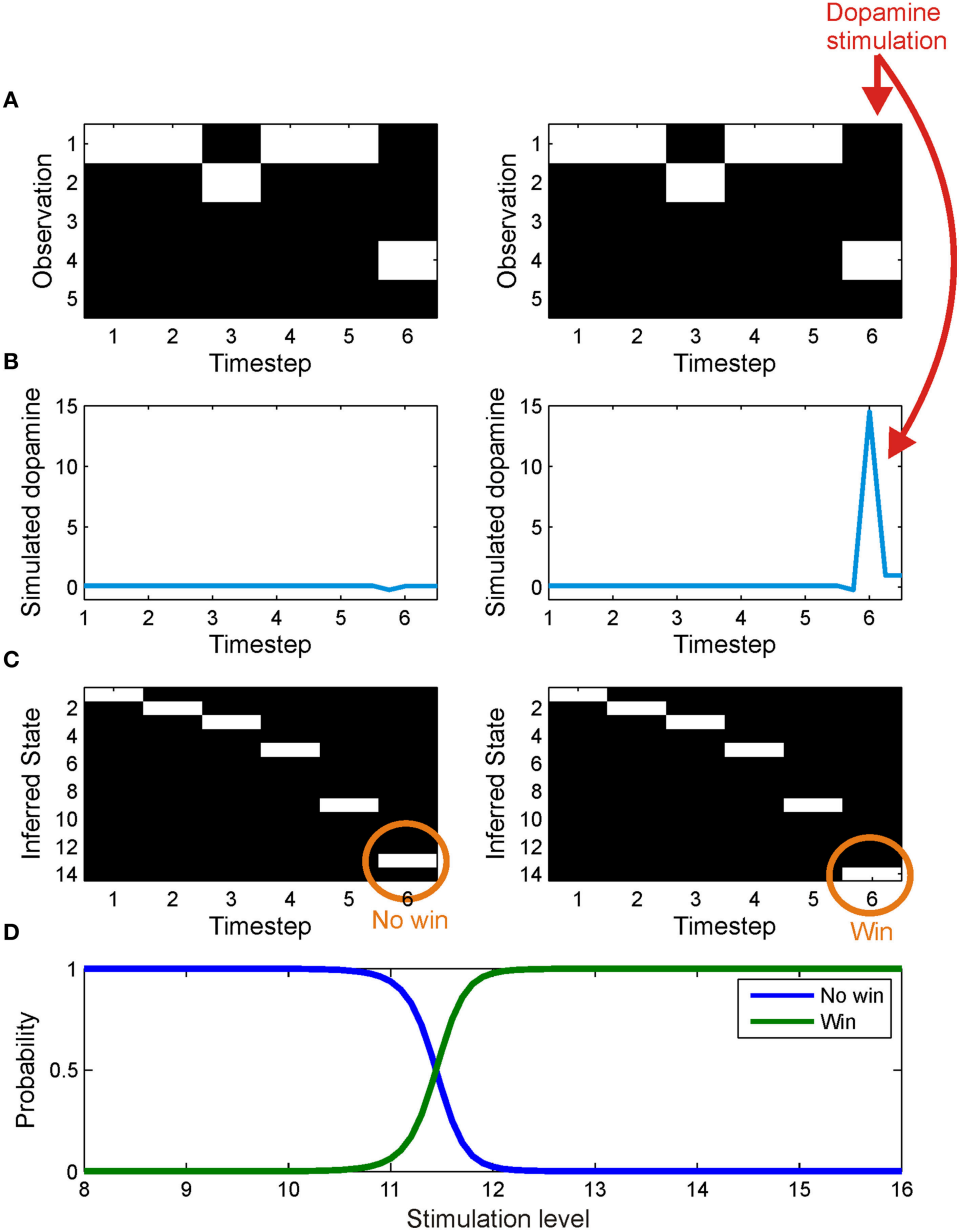
**The effect of simulated stimulation of the dopaminergic midbrain at outcome presentation**. On both trials, the agent was presented with an identical series of observations **(A)**, corresponding to observing cue one and a no win outcome. In one case (left column) the agent was allowed to infer precision as usual, leading to a small dip in precision at outcome time **(B)** and the correct inference that it had reached a no win outcome state **(C)**. In the other trial (right panel), midbrain stimulation was simulated by fixing expected precision at a high value at outcome time(γ_6_ = 16) **(B)**. This leads, via the effect of precision on state estimation (see update Equation 23 and Friston et al., 2013) to an incorrect inference that it has reached a win outcome state **(C)**. **(D)** shows the effect on inference of stimulation with values varying between 8 and 16. The posterior probability of being in a win outcome state (green) increases as stimulation strength increases, whilst the posterior probability of being in a no win outcome state (blue) falls correspondingly.

We simulated the effect of artificially stimulating midbrain dopaminergic neurons at outcome presentation by fixing expected precision at the final epoch of each trial. To demonstrate the effect of artificially increased precision on inference we simulated two trials, using agents with naïve (pre-learning) beliefs as described above (Figure 7). In both cases the same cue (cue one) was presented, the same action (response one) selected, and a no win outcome received. In one simulation, precision was estimated as normal, but in the other it was artificially fixed to a high value at the last state transition $\left({\overset{⌢}{\gamma }}_{6}=16\right)$. To further quantify how inference about hidden states varied with expected precision, we then simulated trials in which precision at the last epoch varied between 8 and 16 in 0.1 intervals (Figure 7).

**Figure 8:**
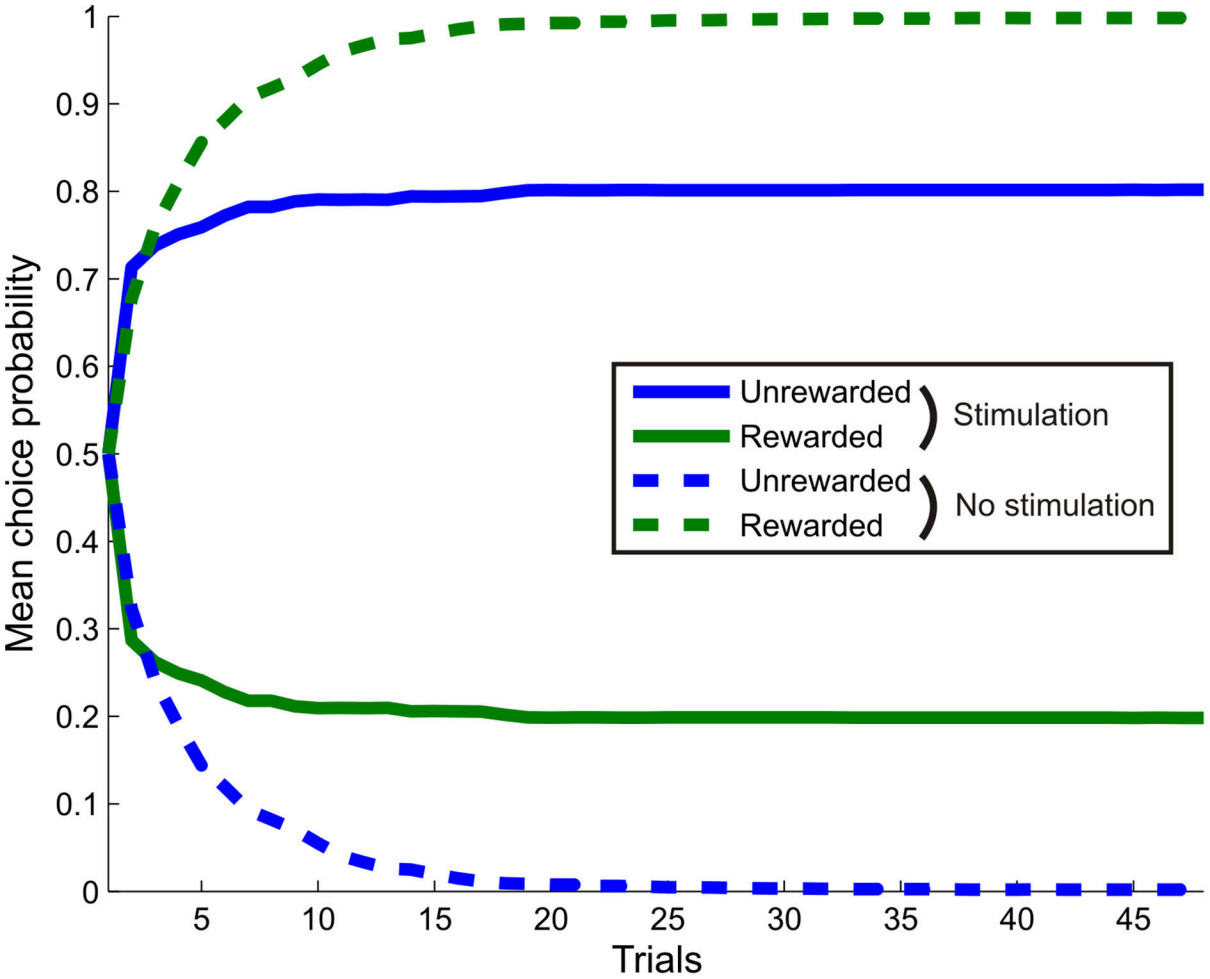
**The effect of simulated stimulation of the dopaminergic midbrain on learning**. The agent was presented with a single cue, with task contingencies such that making response one (blue) never led to reward, whilst response two (green) led to reward with probability 0.5. In the stimulation condition (bold lines), selection of response one was always followed by simulated stimulation at outcome time (γ_6_ = 16). In the control condition (dashed lines), no stimulation occurred. Stimulation was sufficient to induce a reversal in preference, with response one selected more often, even though it was never rewarded. This replicates the findings of recent optogenetic stimulation studies, even though stimulation only affects inference directly, rather than learning. (Choice behavior averaged over 256 repetitions of a 48 trial session).

Having shown that artificially high precision is sufficient to produce aberrant inference, we then explored the effects of this perturbation on learning. To do this, we presented the agent with a single cue, with contingencies such that response two led to reward on fifty percent of occasions, and response one never led to reward, but was reinforced with midbrain stimulation $\left({\overset{⌢}{\gamma }}_{6}=16\right)$. This allowed us to ask whether dopamine-mediated failures of inference are sufficient to explain behavioral capture, even when the alternative behavior is associated with greater reward. We simulated 256 repetitions of a single 48 trial session, and compared choice behavior with that of an agent that was allowed to infer precision normally (Figure 8).

## Results

### Instrumental learning performance

As expected, given its approximate optimality, the agent quickly and accurately learns the experimental contingencies (Figure 4). This learning is accompanied by a progressive minimization of the free energy, indicating a progressive improvement in (the evidence for) its model of the environment. Taken together, this shows that our approximate Bayesian inference scheme is sufficient to enable the agent to learn and behave adaptively (Figure 3) in an uncertain environment.

### Phasic dopamine and the dynamics of expected precision

In keeping with an hypothesis that dopamine encodes expected precision (Friston et al., 2014; Schwartenbeck et al., 2015a), the simulated dopamine dynamics from our model closely resemble classical observations of phasic dopaminergic firing during conditioning (Schultz et al., 1997; Schultz, 1998; Day et al., 2007; D'Ardenne et al., 2008; Flagel et al., 2011; Cohen et al., 2012). More specifically, prior to learning, simulated dopamine responses show no effect at the time of cue or conditioned stimulus (CS) presentation, but a strong modulation at presentation of the outcome or unconditional stimulus (US; Figure 5). After learning however, responses are observed to reward-predicting cues, with responses to the outcome reflecting the difference between expected and observed reward, thus resembling an RPE (Schultz et al., 1997; Figure 5), even though prediction errors are not used for reward learning *per se*(when plotting simulated dopamine time courses here and elsewhere, we remove the simulated responses from the first epoch at time step one, as this shows a boundary artifact as a result of the deconvolution used to transform the expected precision into a simulated dopamine response).

We next examined the evolution of simulated dopaminergic responses during learning. In real data, responses transfer directly from outcomes to cues (Hollerman and Schultz, 1998; Pan et al., 2005) rather than shifting progressively backwards in time as predicted by classical TD models (Schultz et al., 1997; although see discussion) Direct transfer is replicated in our simulated data (Figure 6A), thus resembling dopamine dynamics even at this fine-grained (epoch by epoch) scale.

### Simulating dopamine depletion

Simulating dopamine depletion by fixing precision to an extremely low value $\left(\overset{⌢}{\gamma }=0.1\right)$ appears to impair learning, assessed on the commonly used metric of proportion of correct choices (Figure 6B). However, learning of task contingencies does occur, but this is masked by the effect of low precision on action selection, which becomes largely outcome-insensitive. This is immediately revealed when normal dopamine function (normal precision estimation) is restored (Figure 6B). This effect of dopamine on performance, as opposed to learning, is consistent with findings of a number of studies in both humans (Frydman et al., 2011; Shiner et al., 2012; Smittenaar et al., 2012) and other animals (Berridge and Robinson, 1998; Cannon and Palmiter, 2003; Flagel et al., 2011; Berridge, 2012; Saunders and Robinson, 2012). It is also consistent with the recent finding that transient inhibition of dopaminergic neurons via direct optogenetic stimulation of the lateral habenula reduced rats' tendency to choose preferred options (Stopper et al., 2014).

### Simulating midbrain stimulation

Stimulation of the dopaminergic midbrain, simulated by fixing expected precision at the final time step to 16, was sufficient to induce incorrect (optimistic) inference about the final outcome state (Figure 7). Specifically, despite being exposed to identical observations, the agent inferred that the outcome was a “win,” rather than a “no win” (Figure 7C). This reflects the effect of estimated precision on state estimation (Equation 23). Intuitively, aberrant inference about hidden states occurs because in order to perform optimal inference, the agent has to explain why its estimated precision is so high. The only way to do this, given that precision has been artificially fixed, is to infer that it is actually in a win state, despite sensory evidence to the contrary. In other words, dopaminergic stimulation creates the illusion (or delusion) of a reward that subsequently drives learning. In keeping with this account, we note that administration of dihydroxy-L-phenylalanine (L-DOPA) has been shown to increase optimism (Sharot et al., 2012). This is a nice illustration of the circular dependency among Bayesian estimators that is a necessary feature of variational inference. In this instance, it demonstrates that state estimation (perceptual inference) can be biased by estimated precision, in the same way that expected precision depends upon estimated states.

We next tested whether, as expected, the effects of stimulation on inference also affected learning such that stimulation, even in the absence of reward, is sufficient to capture behavioral responses (Tsai et al., 2009; Rossi et al., 2013). Simulated choice behavior unambiguously demonstrates this. Stimulation induces a reversal in responding such that the agent actually preferred to select an option that never led to reward, over one which led to reward on half the trials (Figure 8). Thus, even though in our model dopaminergic activity does not directly affect learning itself (Schultz et al., 1997), stimulating dopamine neurons is sufficient to drive learning, as observed empirically (Tsai et al., 2009; Adamantidis et al., 2011; Witten et al., 2011; Rossi et al., 2013; Steinberg et al., 2013), via effects of dopamine on inference.

## Discussion

We show that by extending a generic variational scheme for active inference to include learning, it is possible to derive simple and neurobiologically plausible learning rules. These updates enable an agent to optimize its model of response contingencies and behave effectively in the context of a simple instrumental conditioning task. However, in addition to being a model of behavior, our variational scheme constitutes an hypothesis about brain function allowing predictions about neuronal activity that can be compared with empirical data. These predictions can span single unit studies through to functional neuroimaging data (Schwartenbeck et al., 2015a).

We used an implicit process theory to show that changes in predicted dopamine responses, over the course of learning, closely resemble those reported during conditioning (Schultz et al., 1997; Schultz, 1998; Day et al., 2007; D'Ardenne et al., 2008; Flagel et al., 2011; Cohen et al., 2012). Our model also explains the apparently puzzling observation that while dopamine does not seem to be necessary for reward learning (Berridge and Robinson, 1998; Cannon and Palmiter, 2003; Robinson et al., 2005; Robbins and Everitt, 2007; Flagel et al., 2011; Berridge, 2012; Saunders and Robinson, 2012; Shiner et al., 2012; Smittenaar et al., 2012), direct excitation of midbrain dopaminergic cells can substitute for the reinforcing effects of rewards (Tsai et al., 2009; Adamantidis et al., 2011; Witten et al., 2011; Rossi et al., 2013; Steinberg et al., 2013). This establishes it as a plausible account of the role of dopamine in reward learning and action selection, though much development needs to be done to explain a broader range of the multiplicity phenomena in which dopamine is known to play a key role (Collins and Frank, 2014).

In our model, RPE-like dopaminergic responses emerge as a result of learning (Figure 4), rather than being a causal mechanism that drives learning. This provides an explanation for the puzzling fact that while phasic dopamine responses resemble an RPE signal, reward learning can proceed in the absence of dopamine (Figure 6). It also explains why, in some situations at least, dopaminergic manipulations have their greatest impact on task performance rather than reward learning (Shiner et al., 2012; Smittenaar et al., 2012; Eisenegger et al., 2014), as well as for the fact that transient inhibition of dopamine neurons via stimulation of the lateral habenula has been shown to reduce the influence of subjective preferences on action (Stopper et al., 2014). Indeed, given the striking effect of dopamine depletion on performance in our simulated instrumental conditioning task (Figure 6), it is possible that studies that do not explicitly separate the effects of dopamine on performance and learning (Frank et al., 2004; Pessiglione et al., 2006; Moustafa et al., 2008; Rutledge et al., 2009; Voon et al., 2010; Nagy et al., 2012; Chowdhury et al., 2013) may in fact reflect the consequences of changes in expected precision. Additionally, our model can explain putative dopaminergic reward prediction error responses in tasks that involve the deployment of “model-based” schemes that do not depend upon temporal difference learning (Daw et al., 2011; Schwartenbeck et al., 2015a), where task contingencies are explicitly described (and hence must be generated on a trial-by-trial basis) rather than acquired as a product of extensive learning (Rutledge et al., 2010), or when physiological states are manipulated between learning and task performance (Berridge, 2012; Robinson and Berridge, 2013).

Our simulations offer an explanation for the fact that, while not necessary for learning, dopaminergic responses seem sufficient to support learning. This is attested by several recent studies that have used direct optogenetic manipulation of dopaminergic activity to demonstrate effects on conditioned place preference (Tsai et al., 2009), and the acquisition and extinction of reward contingencies (Adamantidis et al., 2011; Witten et al., 2011; Rossi et al., 2013; Steinberg et al., 2013; Stopper et al., 2014). Interestingly, in our model these effects occur due to dopamine's role in optimistically biasing inference (Sharot et al., 2012), rather than reflecting a direct effect on learning (i.e., dopamine stimulation effects learning vicariously through aberrant inference or incentive salience, leading to aberrant associative plasticity). [This does not though rule out an additional effect of dopamine on learning, as predicted by “three-factor” Hebbian learning rules (Reynolds et al., 2001; Collins and Frank, 2014)] Our simulations do, however, highlight the potential pitfalls when interpreting behavioral findings, even those from sophisticated and precisely controlled experiments and the importance of using explicit computational models to understand cognition. It also demonstrates the far-reaching consequences of the simple truism that disturbances in inference lead inexorably to disturbances in learning, something that is likely to be of key importance for understanding psychiatric disorders such as psychosis (Fletcher and Frith, 2008; Montague et al., 2012; Adams et al., 2013; Schwartenbeck et al., 2015b).

Our model makes clear predictions about the evolution of dopaminergic responses during learning. Specifically, it predicts that rather than shifting progressively backwards in time across intervening epochs, responses will transfer directly from outcome to cue (Figure 6A), in keeping with what is observed empirically (Hollerman and Schultz, 1998; Pan et al., 2005). This contrasts with the predictions of classic temporal difference learning accounts of dopamine function (Schultz et al., 1997). It can however, be accommodated within a TD framework by augmenting the basic TD model with eligibility traces (Sutton and Barto, 1998; Pan et al., 2005).

A possible approach to reconciling sufficiency-without-necessity of dopamine for reward learning with the TD account appeals to a distinction between a “model-based” system that employs explicit models of the environment and a “model-free” system that uses simple TD learning (Gläscher et al., 2009; Daw et al., 2011; Dolan and Dayan, 2013; Dayan and Berridge, 2014). On this account, learning still occurs under dopamine depletion as a result of the “model-based” system (Dayan and Berridge, 2014). However, if dopamine is only relevant for a model-free learner, it is difficult to explain why performance is severely impaired in its absence, since an intact model-based system should still be available to guide behavior. The “model-free” vs. “model-based” account is also difficult to square with data from tasks where the use of TD learning is implausible (Rutledge et al., 2010; Schwartenbeck et al., 2015a), or where outcome signals seem to reflect a mixture of “model-free” and “model-based” prediction error signals (Daw et al., 2011). Instead we contend that behavior on these and similar tasks can be understood purely in terms of “model-based” processing implicit in active inference, albeit with hierarchical models of varying complexity (FitzGerald et al., 2014), and that there is little need to suppose the existence of a “model-free” TD learner at all. However, we acknowledge that positing separate “model-based” and “model-free” systems has considerable explanatory power, and that this is a widely influential view within the decision neurosciences (Wunderlich et al., 2012; Lee et al., 2014) and beyond (Gillan and Robbins, 2014; Huys et al., 2015).

The hypothesis that dopamine encodes expected precision has clear affinities with the incentive salience hypothesis of dopamine function (Berridge, 2007) as well as more general ideas relating it to behavioral “activation” (Robbins and Everitt, 2007). In each case, dopamine plays a fundamentally modulatory role, and mediates a sensitivity of behavior to potential reward (Stopper et al., 2014). From this perspective, the main contribution of this and related work (Friston et al., 2014; FitzGerald et al., 2015; Schwartenbeck et al., 2015a) is to formulate key insights from these theories within a formal framework derived under the broader notion of active inference (Mumford, 1992; Dayan et al., 1995; Fletcher and Frith, 2008; Friston, 2010; Clark, 2012).

Our approach to understanding dopaminergic function is primarily “top-down,” in the sense that we seek to understand it in terms of normative theories of brain function. This coexists quite happily with alternative “bottom-up” approaches based on what is known about striatal anatomy and physiology (Frank, 2011; Humphries et al., 2012; Collins and Frank, 2014; Fiore et al., 2014). In future we hope to develop this framework further to bring it more closely in to line with the underlying neurobiology, and at the same time that these ideas prove helpful for interpreting the findings of more biologically grounded modeling approaches. Generally, dopamine has been implicated in an enormous range of behavioral phenomena, and it seems unlikely that any single computational theory will be sufficient to explain them all, particularly given recent findings suggesting greater diversity between midbrain dopaminergic neurons than had previously been believed (Roeper, 2013). Our aim here is to develop one such theory, based on increasingly popular normative approaches to cognition (Tenenbaum et al., 2006; Friston et al., 2013; Pouget et al., 2013; Schwartenbeck et al., 2013), rather than to attribute a single, definitive, function to the dopaminergic system.

In our relatively minimal model, the only free parameters are those governing the gamma distributions over the expected precision parameter, the Dirichlet distributions over the values in the A and B matrices, and the values in the C matrix that determines the agent's preferences (the states it expects to be in). Altering the parameters that govern expected precision will have the consequence of increasing expected precision when the ratio increases, and decreasing it when the ratio decreases. Although we do not explore it here, these parameters can also be learnt, and this provides an attractive way to understand the link between dopamine, average reward and response vigor (Beierholm et al., 2013), since learning will result in larger values of expected precision (which might plausibly translate into speed of responding), when preferred outcomes are more common. Altering the relative sizes of the concentration parameters of the Dirichlet priors over the A and B matrices changes the agent's beliefs about the mapping from hidden states to observations, and transitions between hidden states respectively. These parameters can be thought of, effectively, as the number of outcomes and transitions that have been previously encountered. Changing these parameters nuances learning rates, where larger values, which correspond to beliefs based on a greater number of observations, produce slower learning (because the agent requires more evidence to update beliefs based upon more experience).

In this paper, we have largely effaced the difference between Pavlovian and instrumental conditioning (Dickinson and Balleine, 1994). In part, this reflects the fact that many of the canonical findings we sought to replicate have been reported in the context of instrumental conditioning (Mirenowicz and Schultz, 1996; Schultz et al., 1997; Tsai et al., 2009; Shiner et al., 2012). However, it is also the case that Pavlovian learning tasks require action, at least in the minimal form of consummatory behavior. [In fact overt conditioned behavioral responses is typically a precondition for recording meaningful data (Fiorillo et al., 2003)]. As such, within the framework presented here, the distinction between Pavlovian and instrumental conditioning paradigms can be thought of as reflecting the number and quality of the policies available for selection, rather than anything deeper. However, we acknowledge that the cognitive processes mediating Pavlovian and instrumental learning may be more distinct than this (Dickinson and Balleine, 1994; Dickinson et al., 2000), for example they might depend upon different types of generative model (Dolan and Dayan, 2013; FitzGerald et al., 2014). Under the precision hypothesis, dopaminergic activity is directly linked to action selection rather than learning (Figure 6), this raises the possibility that under appropriate circumstances—those where there truly is no action to perform—dopaminergic responses will cease to track reward, a possibility for which there is some evidence (Guitart-Masip et al., 2011, 2012, 2014).

In formulating our scheme, we make a clear distinction between inference about hidden states and learning about model parameters. In one sense this is arbitrary, as can be seen from the fact that we use exactly the same principles to perform both. However, the fact that parameters are treated as fixed, at least at the time scale of interest, allows evidence to be accumulated across trials, which is crucial for adaptive performance here. Neurobiologically, this is likely to correspond to a distinction between states which are encoded by neuronal firing, and parameters which are encoded by synaptic weights. The consequences of allowing model parameters to vary slowly have been considered in compelling ways elsewhere (Behrens et al., 2007; Mathys et al., 2011; Diaconescu et al., 2014), and it would be interesting to include this within our scheme (by treating parameters as slowly fluctuating states). In general, it seems likely that learning and inference occur at a hierarchy of time scales (Kiebel et al., 2009), and any comprehensive account of cognitive function will need to accommodate this.

The learning rules we derive are simple, and could plausibly be implemented by neuronal circuits (Abbott and Nelson, 2000). This is important as our intention here is to provide a (necessarily simplified) process theory that specifies how inference and learning might be performed by embodied agents. We restrict ourselves however to considering only online learning, and ignore for the present the question of whether additional retrospective offline learning also occurs. We intend to return to this question in future work.

Although our modeling results speak to several well-established findings, there are also a number of phenomena that they fail to explain. One of these is dopamine's key role in modulating the amount of effort that an animal will expend in order to attain a reward (Salamone et al., 2007; Kurniawan, 2011), as well as its (very likely related) role in mediating the vigor of responding (Beierholm et al., 2013). In addition, dopamine seems to be closely linked to action (as opposed to inaction; Guitart-Masip et al., 2014), an asymmetry is not explained by our framework, since doing nothing is simply treated as an extra type of action. To address these issues, future developments of this computational framework are needed, which are likely to involve bringing it more closely in line with anatomically-motivated models that (for example) clearly separate action from inhibition (Frank, 2011). Another key issue that remains to be addressed is the finding that inhibition of dopaminergic neurons can result in aversive conditioning (Tan et al., 2012; Danjo et al., 2014), a phenomenon not presently accounted for by our model.

In conclusion, we have described a theoretical framework for simulating planning and decision-making using active inference and learning. We use this to test an hypothesis that dopamine encodes expected precision over control states by modeling a simple instrumental conditioning paradigm. Strikingly, our model was able to replicate not just observed neuronal dynamics, but also the apparently paradoxical effects of dopamine depletion and midbrain stimulation on learning and task performance. Whilst our proposal has clear kinship with other accounts of the role dopamine plays in learning and motivation [in particular the incentive salience hypothesis (Berridge, 2007)], to our knowledge no other “top-down” theory based on a hypothesized computational role played by dopamine currently accounts for all of the phenomena reproduced here. As such, we believe that this work represents a novel integrative approach to value learning and the role of dopamine in motivated behavior.

## Author contributions

TF and KF created the model. TF implemented the model and performed simulations. TF, RD, amd KF wrote the paper.

### Conflict of interest statement

The authors declare that the research was conducted in the absence of any commercial or financial relationships that could be construed as a potential conflict of interest.
