# Supplementary material for: Dopamine, reward learning, and active inference
Source: Front Comput Neurosci. 2015 Nov 4;9:136. doi: 10.3389/fncom.2015.00136 (PMC4631836; doi:10.3389/fncom.2015.00136)
Supplement: Supplementary file 1 [file DataSheet1.DOCX]

**Appendix**

Let $\tilde{x}=\tilde{s},\tilde{u},\tilde{\gamma},\mathbf{A},\mathbf{B}$ denote the hidden variables and the sufficient statistics of the approximate posterior . Using the dot notation $A\cdot B=A^{T}B$, the variational free energy can be expressed in terms of accuracy and complexity (ignoring constants):

Differentiating the variational free energy with respect to the sufficient statistics gives

Finally, we obtain the variational updates by solving for zero and rearranging:

These now form the basis for the Bayesian belief updates in the main text.
